# Management of cytotoxic chemotherapy in patients undergoing dialysis: a still unresolved issue of onconephrology

**DOI:** 10.1007/s40620-024-02102-7

**Published:** 2024-10-15

**Authors:** Marta Pirovano, Carlo Ganini, Maurizio Gallieni, Camillo Porta, Laura Cosmai

**Affiliations:** 1 Onconephrology Outpatients Clinic, Division of Nephrology and Dialysis, ASST Fatebenefratelli Sacco, Fatebenefratelli Hospital, Piazzale Principessa Clotilde 3, 20121 Milan, Italy; 2 Dana-Farber Cancer Institute, Boston, MA USA; 3 Interdisciplinary Department of Medicine, University of Bari “Aldo Moro”, Bari, Italy; 4 Division of Medical Oncology, A.O.U. Consorziale Policlinico di Bari, Bari, Italy; 5 Department of Biomedical and Clinical Sciences, University of Milan, Milan, Italy; 6 Division of Nephrology and Dialysis, ASST Fatebenefratelli-Sacco, Milan, Italy

**Keywords:** Chemotherapy, Dialysis, Onco-nephrology, CKD, ESRD

## Abstract

**Abstract:**

The incidence of tumors increases significantly in individuals with chronic kidney disease (CKD), particularly among those undergoing dialysis. This dialysis-associated condition not only impacts therapy but also influences the prognosis of oncological patients, contributing to heightened mortality rates related to both cancer and non-cancer causes. Importantly, it stands as a primary factor leading to suboptimal utilization of therapies. Dosage adjustment for many types of chemotherapy is a necessity in patients with kidney impairment. However, due to a lack of comprehensive knowledge about the pharmacokinetic and pharmacodynamic properties of these drugs in dialysis, adjustments are often made empirically, and in many cases, chemotherapy is avoided altogether. In this review, we highlight the current challenges and gaps in knowledge, and emphasize the imperative need for dedicated research to establish evidence-based guidelines for chemotherapy management in this vulnerable patient population.

**Graphical abstract:**

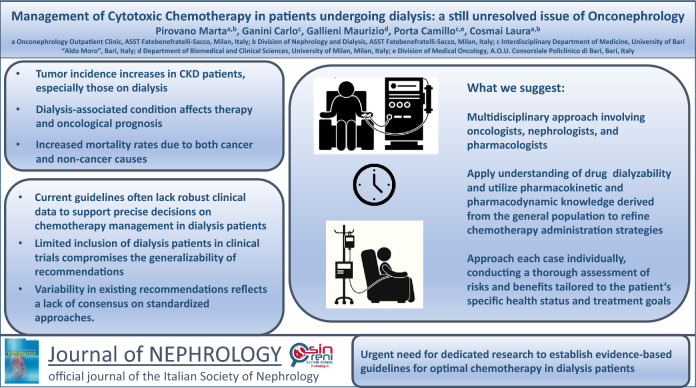

## Introduction

Managing cytotoxic chemotherapy in patients undergoing dialytic treatment poses a complex challenge that requires a careful and multidisciplinary approach.

While numerous papers discuss chemotherapy in dialysis, a lack of uniformity exists concerning the recommended dosage and administration schedules for these drugs.

Available guidelines are often conflicting and should be regarded more as expert opinions than evidence-based guidelines. This is even more problematic since being on dialysis has been, and still is, an almost universal exclusion criterion from clinical trials. Furthermore, real world evidence is hampered by the limited number of patients treated and the extreme variability of doses and schedules of administered chemotherapeutics, again reflecting the lack of consensus on this important matter. Therefore, the need for trials specifically involving this patient population has been advocated by both regulatory Authorities [1, 2], and by experts in the field [3]. These trials would play a critical role in determining the appropriate dosage, administration methods, and timing for chemotherapy in patients on dialysis, ensuring that these individuals receive optimal and tailored treatment. Unfortunately, to date, this call for action has been largely unanswered.

## Cancer in end-stage kidney disease patients: epidemiology

In the late 1990s, it appeared evident that individuals with chronic kidney disease (CKD), and especially those undergoing dialysis, had an elevated risk of developing tumors [4–6].

Since then, a number of epidemiological studies have sought to precisely identify the incidence of cancer in dialysis patients and its main risk factors.

One notable retrospective cohort study, encompassing 482,510 patients, evaluated cancer incidence in patients undergoing chronic dialysis from 1996 to 2009. Of these patients, 37,128 developed cancer within 5 years, indicating a prevalence of 7.65% and a cumulative incidence over 5 years of 9.48%, with kidney, renal pelvis, and bladder cancers being the commonest malignancies diagnosed [7].

These findings were corroborated by an Italian study involving 10,790 hemodialysis patients, revealing a 1.3-fold greater risk of de novo neoplasm compared to the general population, particularly in the initial three years and, even more frequently, within the first year of dialysis [8].

Another study in Oceania assessed the risk of cancer recurrence in dialysis patients, examining 4,912 individuals with a history of neoplasm. Of these, 323 (7%) experienced tumor recurrence, 80% of which were metastatic. Furthermore, 343 patients (7%) developed new neoplasms, with an average time to tumor recurrence from the start of dialysis of 1.2 years and an average onset time for new tumors of 2 years. Survival rates were notably limited, with a 1.3-year average survival for patients with de novo tumors, particularly kidney, urinary tract, and lung cancers. Patients with recurrent disease exhibited a less than 50% 3-year survival, with lymphoma, urinary tract, lung, and melanoma being the most frequently recurrent tumors [9].

This heightened incidence seems linked to shared risk factors between CKD and cancer, including hypertension, age over 65 years, smoking, obesity, alcohol consumption, male gender, cardiovascular disease, and environmental exposure [10, 11]. Additionally, adverse events stemming from kidney failure, such as prolonged exposure to uremic toxins, chronic inflammation, increased infection risk, compromised immune function, malnutrition, and altered DNA repair mechanisms, contribute to this association [8, 12].

Since advancements in dialysis techniques have significantly extended the average lifespan of dialysis patients, this association of long survivorship with an increased risk of developing cancer has ultimately resulted in a larger number of dialysis patients with cancer needing specific treatment.

This underscores the increasing importance of addressing cancer treatment in this patient group [13, 14].

## Cytotoxic chemotherapy across the decades

Cytotoxic chemotherapy was the initial class of drugs employed in treating tumors. In 1942, Louis Goodman and Alfred Gilman administered nitrogen mustards for the first time to a patient with non-Hodgkin’s lymphoma, showcasing that chemotherapy can induce tumor remission [15]. Since then, the use of chemotherapy has expanded significantly, resulting in the proliferation in the number of drugs deployed and the variety of cancers treated. In 1992, the approval of imatinib for chronic myeloid leukemia marked the initiation of the era of molecularly targeted drugs. Although the introduction of new classes of anticancer agents (targeted agents, immune checkpoint inhibitors and antibody–drug conjugates) has improved the survival of cancer patients over the past three decades, cytotoxic chemotherapy persists as a still widely utilized approach today, either as monotherapy or in combination with other chemotherapeutics, or with drugs of different classes (Table 1).

**Table 1:** Chemotherapy in clinical practice [16]

Type of tumor	(Main) Chemotherapeutic agents used	Chemotherapy use over time	Other therapies
Lung	*NSCLC* ADJ/Advanced/metastatic Platinum salts (Cisplatin or Carboplatin in non-*oncogene-addicted* NSCLC) Antifolates (Pemetrexed) Taxanes (paclitaxel and docetaxel) *SCLC* ADJ/Advanced/metastatic Platinum salts (Cisplatin or Carboplatin) Topoisomerase II inhibitors (VP16) Taxanes (paclitaxel and docetaxel)	Overall, slightly decreasing	ICI (Pembrolizumab); ICI (Atezolizumab, Durvalumab) + CT; TA (for *oncogene-addicted* NSCLC, Osimertinib, Afatinib, Crizotinib, Entrectinib, Dabrafenib, etc.)
Colorectal	nADJ/ADJ Platinum salts (Oxaliplatin) Pyrimidine analogues (5-Fluorouracil, Capecitabine) Advanced/metastatic Platinum salts (Oxaliplatin) Pyrimidine analogues (5-Fluorouracil) Topoisomerase I inhibitors (Irinotecan)	Stable	TA (Bevacizumab, Aflibercept, Panitumumab, Cetuximab) + CT, ICI (in MSI-H, Pembrolizumab)
Esophagus/Stomach	nADJ/advanced/metastatic Platinum salts (Oxaliplatin) Pyrimidine analogues (5-Fluorouracil) Topoisomerase I inhibitors (Irinotecan) Taxanes (Docetaxel) Docetaxel	Stable	TA (Trastuzumab in Her2 +) + CT
Pancreas	nADJ/ADJ/Advanced/metastatic Platinum salts (Oxaliplatin) Pyrimidine analogues (5-Fluorouracil) Topoisomerase I inhibitors (Irinotecan) Taxanes (mAB-Paclitaxel)	Stable	TA (Olaparib in BRCAmut tumor) + CT
Ovarian	ADJ/Advanced/metastatic Platinum salts (Carboplatin) Taxanes (Paclitaxel) Topoisomerase I inhibitors (Topotecan) Topoisomerase II inhibitors (Doxorubicin)	Stable	TA (Bevacizumab, Olaparib se BRCAmut tumor) + CT
Endometrium	ADJ/Advanced/metastatic Platinum salts (Cisplatin or Carboplatin) Taxanes (Paclitaxel)	Stable	ICI; TA (Trastuzumab for Her2 + tumors) + CT
Head and neck	ADJ/Advanced/metastatic Platinum salts (Cisplatin or Carboplatin)	Stable	ICI (Pembrolizumab) + CT; TA (Cetuximab)
Urothelial	nADJ/ADJ/Advanced/metastatic Platinum salts (Cisplatin or Carboplatin) Cytidine analogues (Gemcitabine)	Stable	ADC (Enfortumab Vedotin), ICI (Pembrolizumab, Nivolumab)
Breast	nADJ/ADJ/Advanced/metastatic Platinum salts (Cisplatin or Carboplatin) Cytidine analogues (Gemcitabine) Topoisomerase II inhibitors (Doxorubicin) Alkylating agents (Cyclophosphamide) Taxanes (Paclitaxel, nAB Paclitaxel)	Heavily decreasing	ADC (Sacituzumab Govinotecan, Trastuzumab Deruxtecan), TA (Trastuzumab, Pertuzumab, CDK4/6 inhibitors, PARP inhibitors, Talazoparib, Everolimus)
Prostate	Metastatic (CRPC) Taxanes (Docetaxel, Cabazitaxel)	Increasing	TA (ARSI, Olaparib in BRCAmut tumors)
Sarcoma	nADJ/ADJ/Advanced/metastatic Topoisomerase II inhibitors (Doxorubicin) Alkylating agents (Ifosfamide)	Stable	TA (Larotrectinib, Entrectinib for NTRK fusion tumor)
Lymphoma/Leukemia	Alkylating agents (Cyclophosphamide) Vinca alkaloids (Vincristine), Topoisomerase II inhibitors (Doxorubicin, Daunorubicin)	Stable	CT + TA (Imatinib, Dasatinib, Nilotinib, Obinutuzumab, Rituximab, Pemigatinib, etc.)
Multiple Myeloma	Alkylating agents (Melphalan)	Stable	ADC (Belantamab mefodotin); Proteasome inhibitors (bortezomib, Carlfizomib); Immunomodulatory agents (thalidomide, lenalidomide, pomalidomide), Anti CD38 (Daratumumab)

*NSCLC* non-small cell lung cancer, *SCLC* small cell lung cancer, *nADJ* neoadjuvant treatment, *ADJ* adjuvant treatment, *ICI* immune checkpoint inhibitors, *CT* chemotherapy, *TA* targeting agents, *MSI-H* microsatellite instability high, *ADC* antibody–drug conjugate

The management of chemotherapy in patients undergoing dialysis remains a significant challenge for both clinical oncologists and nephrologists. This is attributed to the fact that many chemotherapy drugs, even those with only partial renal excretion, are to a certain extent, eliminated by the kidneys, and dialysis can alter the metabolism of the majority of drugs, including those with reduced or no renal excretion [17].

In this realm, the primary challenges lie in determining the appropriate drug dosage and the timing of administration with regard to the dialysis session. Optimizing both parameters is crucial. On one hand, administering a standard dose of an active drug that relies mainly on the kidneys for clearance can result in overexposure to the toxic substance, leading to increased adverse events and, possibly, a decrease in survival. On the other hand, inappropriate reduction in the dose of a drug excreted through bile or feces, or its premature removal from the bloodstream by dialysis, may result in undertreatment, consequently diminishing its effectiveness [18].

## Inclusion of individuals with end-stage kidney disease in clinical trials for cancer therapy

The inclusion of individuals with end-stage kidney disease (ESKD) or on dialysis in clinical trials for cancer therapy is a vital step toward ensuring that this patient population receives equitable access to innovative treatments. Historically, such individuals have been underrepresented in clinical trials, leading to a gap in understanding the efficacy and safety of cancer therapies specifically tailored to their unique medical circumstances.

Phase I and II clinical trials typically involve individuals with normal or mildly reduced kidney function. The problem persists into phase III studies, where only a subset of patients with moderately decreased glomerular filtration rate (GFR) are included. Consequently, data related to patients with severely decreased estimated GFR ([eGFR] < 30 ml/min/1.73 m^2^), kidney failure, or those undergoing dialysis are notably scarce or completely absent before drug approval. Due to limited data and lack of experience concerning the safety of drugs use in these populations, technical data sheets often state that the drug is contraindicated in patients with advanced kidney failure [19].

To address this issue, regulatory bodies such as the European Medicines Agency (EMA) and the Food and Drug Administration (FDA) have issued specific recommendations that encourage the inclusion of CKD patients in clinical trials, outlining defined methods for estimating GFR and establishing drug dose adjustments [1, 2].

Nonetheless, a 2018 study by Kitchlu et al. highlighted that, of 310 clinical trials analyzed from 2012 to 2017 regarding oncologic drugs for the treatment of the five most common tumors (bladder, breast, colorectal, lung, and prostate), 85% categorically excluded patients with CKD, and 100% excluded patients on dialysis [20]. Similarly, a retrospective study in 2022 by Delaye et al., evaluating the percentage of phase 3 clinical trials on systemic oncology drugs excluding patients with CKD, found that 68% (185 trials) had at least one exclusion criterion based on kidney function [21].

These findings are significant considering the above-mentioned high incidence of tumors in patients with CKD. The exclusion of such patients from trials means that clinicians are unsure how to navigate dosing when the pharmaceutical company lists CKD as a contraindication, or mentions no studies in that population. As a result, these patients are sometimes not considered for oncological treatments.

Importantly, patients with CKD are not only excluded from trials involving drugs with high nephrotoxic potential or a high risk of adverse events due to reduced renal excretion, but also from trials in which the kidney plays a less decisive role in pharmacokinetics and pharmacodynamics [20].

Addressing this gap is essential; by including patients with ESKD or on dialysis, researchers can gather valuable data to tailor therapies that take into consideration the specific needs and challenges associated with severe kidney impairment.

In 2020, Sprangers et al. proposed measures to enhance cancer care for patients with kidney failure. They specifically recommended that national and international regulatory authorities, such as the FDA and the EMA, mandate the inclusion of patients with CKD in clinical trials of drugs, or generate distinct but specific clinical data regarding patients with kidney failure before drug approval. Additionally, they urged carrying out secondary studies on patients with CKD or ESKD following drug approval. Their suggestions included involving nephrologists in the early stages of clinical drug development (and beyond), and organ dysfunction teams to boost the recruitment of CKD patients [22]. They also advocated the establishment of national groups to elevate awareness of this issue [23].

Similar proposals were presented by Delaye et al., who suggested the creation of a method for estimating kidney function that could be applied across all clinical trials. They proposed setting dose modifications for patients with reduced GFR in the initial phases of trials to facilitate the management of CKD patients in later phases, and to prevent the exclusion of ESKD and dialysis patients from clinical trials for drugs that might pose a risk based on pharmacokinetic and pharmacodynamic characteristics [21]. More drug trials inclusive of CKD patients would enable a better understanding of the pharmacokinetics and pharmacodynamics in patients on dialysis.

Currently, clinical data on the use of chemotherapy drugs in dialysis primarily come from case reports, case series, or small retrospective studies. These studies encompass patients treated with a variety of drugs, making it challenging to draw statistically significant and homogeneous conclusions regarding the optimal drug dosage, timing of administration with regard to dialysis sessions, type of dialysis, safety and efficacy profile, and fluid administration [24–28].

The Candy study [29], a retrospective multicenter investigation conducted from 1997 to 2010 on 178 hemodialysis patients, aimed to provide insights into the management of oncological drugs in patients on dialysis who developed tumors after initiating dialysis (with an average time between dialysis initiation and cancer diagnosis of 2.6 years). Among the 178 patients, 50 underwent chemotherapy, resulting in a total of 96 prescriptions involving 36 different drugs. Of these prescriptions, 45% required dose adjustments based on limited literature data or were drugs for which no recommendations were available for dialysis patients. Notably, 75% of the prescribed drugs were administered after the dialysis session. Among the 50 patients treated, 72% received at least one drug needing dose adjustment or lacking recommendations for dialysis patients. Overall, the study revealed that 88% of patients treated with oncology drugs required specific management of the dose or timing of administration for at least one drug [29], although no specific guidelines were available.

However, this study has notable limitations. It lacks guidance on how to adjust the drug dose (only indicating need for adjustment), provides no data on patients treated with reduced doses, and relies on information about the need for dose adjustment or dialyzability derived from individual clinical cases or small case series due to the absence of high-level evidence in this patient setting. Moreover, and perhaps even more importantly, the study lacks data on the response to therapy [29].

## Navigating the distinctions: recommendations or guidelines

In 2017, the Italian Association of Medical Oncology (AIOM) and the Italian Society of Nephrology (SIN) jointly published "Recommendations" on the management of chemotherapy in dialysis patients. Acknowledging that the data available in the literature primarily consisted of single case reports or small case series, the authors attempted to provide guidance on the use of the main chemotherapy drugs. However, the lack of clinical studies on the topic prevented the formulation of evidence-based guidelines, making the recommendations reliant on the limited available evidence [30], and on expert opinion.

In 2022 and in 2024, 2 other reviews aimed to consolidate indications on drug management in cancer patients undergoing chronic dialysis. Once again, the scarcity of clinical studies compelled the authors to base their recommendations on a limited case series, largely overlapping with the ones utilized in the previous review [17, 31].

Some studies have also been published regarding hematological patients, and in 2016, the International Myeloma Working Group published recommendations for the diagnosis and management of myeloma-related renal impairment. Given the high incidence of acute kidney failure requiring dialysis treatment in patients with multiple myeloma, there is some experience in managing cytotoxic chemotherapeutic agents in this setting, in patients undergoing dialysis [32].

One review of the literature focused on patients undergoing peritoneal dialysis. This review comprised 16 case reports involving a total of 18 therapeutic regimens, including 15 chemotherapeutics, 3 molecularly targeted drugs, and no immunotherapy. The Authors concluded that “…elimination of anti-cancer agents seems often altered in such patients, resulting in serious adverse events…”, and thus suggested simply adjusting the dose of each drug [33].

In 2022, the "International Consensus Guideline for Anticancer Drug Dosing in Kidney Dysfunction (ADDIKD)" was published, aiming to offer general guidance on estimating kidney function in cancer patients and utilizing this information to adjust the doses of anticancer drugs. Specific recommendations are provided on administering individual drugs based on the patients' kidney function, considering the severity of adverse events, the compounding effect of other nephrotoxic agents being co-administered, the frailty of the patient, and intent of the anticancer therapy. However, these guidelines do not cover dose adjustments for subsequent therapy cycles nor do they offer specific information on dose adjustments for patients with a GFR below 15 ml/min/1.73 m^2^ undergoing conservative therapy or dialysis. For these patient categories, a multidisciplinary approach involving nephrologists, oncologists/hematologists, and pharmacologists is recommended. The absence of specific clinical studies continues to hinder the development of clear guidelines for managing chemotherapy in the dialysis patient population [34].

In 2022, the Onconephrotoxin Library Collaboration (OLIC) was launched online. This comprehensive database is easily accessible to nephrologists, providing insights into cutting-edge therapies for cancer patients. The aim is to enhance awareness of potential interactions and complications while fostering improved collaboration and communication across medical specialties. It was developed by a consortium of nephrologists, pathologists, and pharmacists in collaboration with the American Society of Onconephrology (ASON), and involved an extensive literature review of clinical trials, scientific papers, and case reports [35].

Since all the new indications published in recent years are consistently based on clinical case reports rather than new clinical studies, the resulting recommendations are often incomplete, if not entirely absent, for specific drugs.

As a whole, contradictions between different papers and recommendations are quite evident, as illustrated in Table 2.

**Table 2:** Recommendations regarding the management of different chemotherapeutic agents in patients undergoing dialysis

		ACKD 2022 [17]	Onco targets and therapy 2023 [28]	AIOM-SIN recommendations [30]	ADDIKD [34]	OLIC [35]
5-Fluorouracil	*Dose*	Not recommended	Standard dose	Standard dose	Consult a multidisciplinary team consisting of oncologists/ hematologists with nephrology and/or clinical pharmacologists	Standard dose or 25% reduction
	*Time*		After HD or N-HDd	After HD		After HD
Capecitabine	*Dose*	Not recommended	Not mentioned	Limited data No data	‘’	Not recommended
	*Time*					
Carboplatin	*Dose*	Tot dose = (target AUC) x (GFR + 25) [Calvert Formula]	Tot dose = (target AUC) × 25 [Calvert Formula considering GFR = 0]	Tot dose = (target AUC) × 25 [Calvert Formula considering GFR = 0]	‘’	PD 75% dose reduction NM-HD
	*Time*	Post HD	Non-dialysis day or 12–24 h before HD	Non-dialysis day		12–24 h before HD
Cisplatin	*Dose*	25–50 mg/m^2^	Reduction 25–50%	Reduction 50–75%	‘’	50% reduction
	*Time*		After HD	After HD		Post HD or N-HDd
Cyclophosphamide	*Dose*	Reduction 25%	Standard dose	Reduction 25%	‘’	NM-HD
	*Time*		After HD or N-HDd	After HD		Post HD or N-HDd
Docetaxel	*Dose*	Not mentioned	No dose adjustment	65 mg/m^2^	‘’	Not mentioned
	*Time*		Before or after HD	Before or after HD		
Doxorubicin	*Dose*	Not mentioned	No dose adjustment	Standard dose	‘’	Not mentioned
	*Time*		After HD or N-HDd	After HD		
Epirubicin	*Dose*	Not mentioned	Standard dose	Standard dose	‘’	Not mentioned
	*Time*		After HD or N-HDd	After HD		
Etoposide	*Dose*	Not mentioned HD	Reduce by 50% or standard dose	Reduction of 40–50%	‘’	NM-HD
	*Time*		Before or after HD	Before or after HD		
Gemcitabine	*Dose*	Non mentioned	Standard dose	Standard dose	‘’	NM-HD
	*Time*		6–12 h before HD	6–12 h before HD		6–12 h before HD
Ifosfamide	*Dose*	Dose reduction to 1.5–2 mg/m^2^	Not recommended	Not recommended	‘’	Not recommended
	*Time*					
Irinotecan	*Dose*	50 mg/m^2^	Standard dose	Reduction dose: 50 mg/m^2^/week	‘’	50 mg/m^2^ weekly or avoid use
	*Time*	After HD or N-HDd	Before HD	After HD		After HD or N-HDd
Methotrexate	*Dose*	Not mentioned	Standard dose	Not mentioned	‘’	Avoid use or 75% reduction
	*Time*		Before HD			
Oxaliplatin	*Dose*	Not mentioned	Reduction of 30%	Reduction of 30%	‘’	50% reduction
	*Time*		After HD or N-HDd	After HD		After HD
Paclitaxel	*Dose*	Not mentioned	Standard dose	Standard dose	‘’	NM-HD
	*Time*		Before or after HD	Before or after HD		
Pemetrexed		Not mentioned	Not recommended		‘’	NM-HD
Vinorelbine	*Dose*	Not mentioned	Not mentioned	Reduction of 25–33%	‘’	Not mentioned
	*Time*			After HD		

*HD* hemodialysis, *GFR* glomerular filtration rate, *N-HDd* non-hemodialysis day, *NM-HD* not mentioned dose reduction for hemodialysis, *AUC* area under the curve, *GFR* glomerular filtration rate, *hrs* hours

Moreover, the exclusion of ESKD patients and those on dialysis from clinical trials hinders the acquisition of data on the pharmacokinetics and pharmacodynamics in these individuals. Even in reported case studies, these drug characteristics are often not investigated. This complicates drug utilization in this population, consequently often leading to a nihilistic approach to these patients.

In the general population, pharmacodynamic properties result from drug interaction with cellular receptors/targets and subsequent downstream pathway activation, while pharmacokinetics can be described by the ADME processes: absorption, distribution, metabolism, and excretion [17]. In ESKD or dialysis patients, modifications can occur for drugs that are not primarily eliminated by the kidneys, but whose active or toxic metabolites are. Additionally, uremic toxins can alter liver enzymes involved in drug metabolism, while in other cases, the liver takes on a greater role in the clearance of certain drugs when kidney function is impaired [17].

In dialysis patients, pharmacokinetics and pharmacodynamics also rely on specific dialysis variables such as ultrafiltration, membrane type and surface, dialysis rate and duration, molecular weight of drugs, and protein binding, among others [36, 37]. These factors require consideration but do not necessarily preclude the administration of chemotherapy during dialysis.

## Clinical practice

In everyday clinical practice, despite overall feasibility, administering systemic anticancer therapy to dialysis patients remains an immense challenge for clinicians, resulting in its infrequent use. A retrospective study by Minegishi et al. illustrated this challenge, indicating that out of 158 oncology patients with lung cancer on dialysis across 22 Japanese hospitals, only 91 received chemotherapy, while 67 received supportive care alone, irrespective of the tumor stage [38]. French data from the CANDY study [29] reported that only 28% of dialysis patients diagnosed with a new neoplasm received systemic oncological therapy, and a Japanese study indicated an even lower percentage (15%) [39].

The tendency toward undertreatment is even more pronounced in the adjuvant setting, which is a dramatic finding given the curability potential of adjuvant treatments. Ishii et al. recently published data from a retrospective study involving 99,761 patients who underwent curative surgery for colon, lung, or breast cancer in Japanese hospitals, including 1% who were on dialysis. The study confirmed that dialysis patients are less likely to undergo adjuvant therapy compared to non-dialysis patients (24% vs. 63%, *p* < 0.001). Furthermore, when adjuvant therapy is initiated, it tends to be shorter in duration for dialysis patients (138 vs. 154 days, *p* < 0.001). The regimens are often modified without consistency, and unnecessary reductions in drug doses are frequent (92% of patients treated in this study vs. 72% in previous studies) [40].

Establishing robust pharmacovigilance practices and maintaining meticulous documentation of chemotherapy administration (dose and time) and of any adverse events is crucial for ongoing patient management and future treatment decisions (Table 3).

**Table 3:** Practical recommendations for managing cytotoxic chemotherapy in dialysis patients

Practical tips
How to Select a Patient for Cytotoxic Chemotherapy
Assess the patient's overall health status (comorbidities, nutritional status, tumor stage, etc.)
Evaluate the patient’s therapeutic goal (curative or palliative chemotherapy)
Consider the availability of alternative drugs with comparable efficacy but lower risks in dialysis patients
Determine the patient’s willingness to proceed after providing appropriate information on the risks and benefits of the treatment
How to Set Up Oncological Treatment
Consider the patient’s general health condition and therapeutic goals when choosing the drug dosage
Consult with a pharmacologist and review literature data to evaluate pharmacokinetics and pharmacodynamics in the general population and any expected differences in dialysis patients
Assess the dialysis method, the membrane used, and the treatment frequency, and determine with the pharmacologist if this combination is optimal for the patient
General recommendations
When possible, especially for fragile patients, and when dealing with drugs considered at high-risk for dialysis patients or for which data are lacking, hospitalization prior to administering at least the first cycle and maintaining close follow-up is recommended
If possible, and in consultation with the pharmacologist, drug blood concentration should be measured in the days following administration, before and after dialysis sessions, to gather as much information as possible for subsequent patient management and for other patients

## Adverse events

Another unique challenge posed by the utilization of chemotherapy in dialysis patients is the critical importance of understanding and managing adverse events associated with various treatment modalities. The unique physiological changes associated with dialysis, coupled with the potential alterations in drug metabolism and elimination, require oncologists and nephrologists to collaborate closely.

Dosing adjustments are pivotal to mitigate adverse effects. Kuo et al. delved into dosing complexities, emphasizing the challenges of dealing with severe toxicity at higher doses and potential efficacy reduction with lower doses. Notably, the study highlights adverse events linked to myelosuppression, leading to dose adjustments and regimen changes. The intricate balance between maintaining efficacy and minimizing toxicity, particularly in the absence of substantial clinical trial data for dialysis patients, adds an additional layer of complexity [27].

Regular monitoring of hematologic parameters and potential organ toxicities becomes paramount to detecting and addressing toxicities promptly. Zhang et al. contribute valuable insights into drug-specific risks, emphasizing the heightened vulnerability of dialysis patients to myelotoxicity induced by cisplatin and carboplatin, the increased risks of cardiotoxicity with doxorubicin and epirubicin, and the potential for hemorrhagic cystitis with cyclophosphamide and ifosfamide. These findings underscore the need for careful risk stratification and individualized treatment approaches [28].

Furthermore, considering alternative chemotherapy regimens with a more favorable toxicity profile for dialysis patients, while ensuring optimal therapeutic efficacy, is a crucial aspect of managing toxicity. Yasuda et al. focused on this aspect; they underscore the potentially harsh impact of CVP (cyclophosphamide, vincristine, and prednisone) and CHOP (cyclophosphamide, doxorubicin, vincristine and prednisone)-like therapies, with or without rituximab, in dialysis patients. This prompts a crucial consideration of alternative drugs like chlorambucil and ibrutinib where there is a choice of treatment, as is the case in indolent Non-Hodgkin lymphomas, signaling the need for more extensive data to guide treatment decisions [24].

## Survival after therapy

Cancer patients with concomitant CKD have worse outcomes compared to those with normal kidney function [20]. Additionally, dialysis patients face a 1.5- to 2.9-fold higher risk of cancer-related mortality than the general population [40]. Furthermore, a study by Minegishi et al. did not clearly demonstrate survival benefits in treating dialysis patients with chemotherapy, suggesting the need for careful evaluation and patient selection before initiating treatment in these individuals [38].

The deteriorating outcomes in these patients can be attributed to the numerous comorbidities prevalent in dialysis patients. However, a significant issue appears to be the under-treatment or suboptimal treatment of patients. Many patients on dialysis are often denied appropriate treatment due to an already mentioned nihilistic attitude, not only among oncologists but also, regrettably, among nephrologists. This hesitancy stems from a lack of comprehensive knowledge of the pharmacokinetics and pharmacodynamics of these drugs in dialysis, coupled with the challenge of synchronizing oncology therapy with dialysis sessions.

Studies on patients with CKD have indicated that appropriately adjusting drug doses based on kidney function before commencing treatment leads to a better oncological prognosis compared to adjusting doses during therapy [18, 41]. While similar studies on dialysis patients are currently lacking, it is reasonable to assume that the trend in terms of prognosis remains consistent.

## Multidisciplinary team

Multidisciplinary coordination between oncology, nephrology, and pharmacology teams, along with continuous pharmacovigilance practices, contributes to enhanced understanding of the nuanced challenges posed by chemotherapy toxicity in the context of dialysis. The evolving landscape of evidence and ongoing research endeavors to emphasize the need for a personalized and multidisciplinary approach in managing adverse events and ensuring optimal outcomes for this unique patient population. The multidisciplinary team should ideally include a Nephrologist and a Pharmacologist who serve as primary contacts for the Oncologists and who, over time, develop expertise and mutual trust with the other team members. Additionally, the involvement of these specialists is critical in the decision-making process, as their unique insights into kidney function and pharmacokinetics can guide the safe and effective administration of chemotherapy in dialysis patients. When these specialists are not present within the hospital, consultation with nephrologists and pharmacologists experienced in the field should be sought to deal with complex clinical cases such as the management of chemotherapy in dialysis patients. Such consultations should be a standard part of the decision-making protocol, ensuring that each patient’s treatment plan is tailored to their specific needs and circumstances. By expanding the multidisciplinary approach, the team can better navigate the complexities of chemotherapy administration in this vulnerable patient population, ultimately leading to improved patient outcomes.

## Ethical aspects

In the realm of chemotherapy for patients undergoing chronic dialysis, the paramount importance of patient education and shared decision-making cannot be overstated. A holistic approach involves engaging patients in comprehensive discussions that highlight the potential risks and benefits of chemotherapy within the specific context of chronic dialysis. These discussions, conducted with the utmost clarity, should consider the patient's overall health status, his/her values and will, his/her quality of life and align with treatment goals. By fostering open communication and ensuring a shared understanding, healthcare providers empower patients to actively participate in decisions regarding their treatment journey.

Navigating the terrain of chemotherapy for individuals on chronic dialysis requires a keen awareness of ethical considerations that go beyond the medical realm. Healthcare providers must delve into discussions around treatment futility and the broader impact of aggressive chemotherapy on the patient's quality of life. In this ethical exploration, it becomes imperative to involve not only the patient, but also his/her family, and the broader healthcare team. This collaborative approach ensures that decisions are made collectively, considering the unique values, preferences, and perspectives of all stakeholders. By acknowledging the ethical dimensions of treatment choices, healthcare providers can strive to align interventions with the overarching goal of enhancing the patient's well-being and preserving their dignity throughout the treatment journey.

## Conclusions

Critically analyzing the available literature on chemotherapy in dialysis reveals a lack of uniformity regarding doses and schedules, as well as of administration times, further complicated by the contradictory information that emerges when comparing different recommendations. Indeed, presently, there are no concrete guidelines to rely on; rather, recommendations (with all the above limits), and expert opinion dominate the landscape. What is dramatically lacking, in our opinion, are dedicated clinical trials in Onconephrology; such trials are crucial for determining the optimal dosage, administration schedules, and overall management of chemotherapy in patients undergoing dialysis. Thus, pharmacokinetic and pharmacodynamic properties of anticancer agents in dialysis should be further investigated and taken into account in order to draw up real guidelines in this peculiar setting. Furthermore, in order to gather sufficient data, it would be necessary to establish a widespread data collection effort, including the creation of a registry to record detailed information on all dialysis patients receiving oncological treatment. Achieving meaningful answers will only be possible through international multicenter collaboration.

In conclusion, the approach to treating cancer in a patient on dialysis should follow that of a patient not on dialysis; this involves conducting thorough assessments of the drug's renal clearance, dosage, and dualizability [29].

As a whole, whether on dialysis or not, patients potentially benefiting from active oncological treatment should be treated, while those not suitable for active treatment (and dialysis, per se, is not an exclusion criterion) should not.

Moreover, ethical considerations, oncological prognosis, quality of life, and the patient's will, values and objectives must be carefully taken into account.

This comprehensive approach would ensure a patient-centered strategy for managing cancer in individuals undergoing dialysis.

## Data Availability

Data sharing not applicable to this article as no datasets were generated or analyzed during the current study.
